# Effects of infant weight gain on subsequent allergic outcomes in the first 3 years of life

**DOI:** 10.1186/s12887-017-0890-0

**Published:** 2017-06-02

**Authors:** Evelyn Xiu-Ling Loo, Anne Goh, Izzuddin Bin Mohd Aris, Oon Hoe Teoh, Lynette Pei-Chi Shek, Bee Wah Lee, Yiong Huak Chan, Mya Thway Tint, Shu-E Soh, Seang-Mei Saw, Peter Gluckman, Keith M Godfrey, Yap-Seng Chong, Fabian Yap, Michael S Kramer, Hugo Van Bever, Yung Seng Lee

**Affiliations:** 1 Singapore Institute for Clinical Sciences (SICS), Agency for Science, Technology and Research (A*STAR), Singapore, 117609 Singapore; 2 Department of Paediatrics, Allergy service, KK Women’s and Children’s Hospital, Singapore, 229899 Singapore; 3 Department of Paediatrics, Yong Loo Lin School of Medicine, National University of Singapore, Singapore, 119228 Singapore; 4 Department of Paediatrics, Respiratory Service Medicine, KK Women’s and Children’s Hospital, Singapore, 229899 Singapore; 5 Biostatistics Unit, Yong Loo Lin School of Medicine, National University of Singapore, Singapore, 119228 Singapore; 6 Saw Swee Hock School of Public Health, National University of Singapore, Singapore, 117549 Singapore; 7 Liggins Institute, University of Auckland, Auckland, 1023 New Zealand; 8 NIHR Southampton Biomedical Research Centre, University of Southampton and University Hospital Southampton NHS Foundation Trust, Southampton, SO16 6YD UK; 9 Department of Obstetrics & Gynaecology, Yong Loo Lin School of Medicine, National University of Singapore, Singapore, 119228 Singapore; 10 Department of Paediatric Endocrinology, KK Women’s and Children’s Hospital, Singapore, 229899 Singapore; 11 Division of Endocrinology and Diabetes, Khoo Teck Puat-National University Children’s Medical Institute, National University Hospital, National University Health System, Singapore, 119074 Singapore; 12 Medical Research Council Lifecourse Epidemiology Unit, Southampton, SO16 6YD UK; 13 Khoo Teck Puat-National University Children’s Medical Institute, National University Hospital, National University Health System, Singapore, 119228 Singapore; 14 Department of Pediatrics and of Epidemiology, Biostatistics and Occupational Health, McGill University Faculty of Medicine, Montréal, QC H3A 1A2 Canada

**Keywords:** Obesity, Allergy, Allergen sensitization, Birth cohort, Early childhood

## Abstract

**Background:**

The association between early weight gain and later allergic outcomes has not been well studied. We examined the relation between weight gain and the subsequent development of allergic outcomes in the first 36 months of life in a Singapore birth cohort.

**Methods:**

In repeated visits in the first 15 months, we measured infant weight and administered questionnaires ascertaining allergic outcomes. At ages 18 and 36 months, we administered skin prick tests (SPTs) to inhalant and food allergens.

**Results:**

At 18 months, 13.5% had a positive SPT, 3.5% had wheeze and a positive SPT, 3.9% had rhinitis and a positive SPT, and 6.1% had eczema and a positive SPT. Higher weight gain from 6 to 9 months, 9 to 12 months and 12 to 15 months were independently associated with a reduced risk of developing a positive SPT at 18 months (p-trend ≤0.03). At 36 months, 23.5% had a positive SPT, 11.9% had wheeze and a positive SPT, 12.2% rhinitis and a positive SPT, and 11.5% eczema and a positive SPT. Higher weight gain from 12 to 15 months was associated with a reduced risk of developing a positive SPT at 36 months (p-trend <0.01). No significant associations were observed between weight gain in any period and wheeze, rhinitis or eczema combined with a positive SPT at 18 or 36 months.

**Conclusion:**

Higher weight gain in the first 15 months of life was associated with a reduced risk of allergen sensitization, but not with combinations of allergic symptoms.

**Trial registration:**

NCT01174875 Registered 1 July 2010, retrospectively registered.

**Electronic supplementary material:**

The online version of this article (doi:10.1186/s12887-017-0890-0) contains supplementary material, which is available to authorized users.

## Background

Allergic diseases and childhood obesity have increased in parallel worldwide in recent decades, suggesting a potential causal link between them [1]. Obesity is also considered a state of chronic inflammation, with activation of multiple cytokines [2]. Positive associations between obesity and allergic diseases in childhood have been reported, [3–5] although studies of the association between weight gain and atopy have shown inconsistent results. [6, 7].

Most previous studies have focused on older children (≥3 years), [7, 8] and knowledge is limited on the impact of early weight gain on subsequent allergic sensitization and atopic conditions (e.g., eczema, asthma, and rhinitis). [9] We hypothesized that rapid weight gain during infancy would be associated with an increased risk of developing allergic outcomes later in childhood and tested this hypothesis in the Growing Up in Singapore Towards healthy Outcomes (GUSTO) birth cohort. To our knowledge, ours is the first study to examine the effect of weight gain in early life on allergic outcomes in an Asian population. This population has a dissimilar genetic constitution to Western populations, along with many differences in dietary and environmental exposures.

## Methods

The methodology of the GUSTO study has been described previously. [10, 11] Briefly, we recruited 1247 healthy pregnant mothers who agreed to enroll their offspring for future follow-up. Interviewers gathered information on demographics, family history of allergy, social data and lifestyle factors. Anthropometric measurements were carried out in the home at 3 weeks and 3, 6, 9, 12 and 15 months of age, with examination of the child at the study clinic site at 18 and 36 months. Definitions were standardized in the questionnaires administered at 3, 6, 9, 12, 15 18, 24 and 36 months to ensure consistency during interviews and home visits. Skin prick testing (SPT) to inhalant allergens (house dust mites *Dermatophagoides pteronyssinus*, *Dermatophagoides farinae,* and *Blomia tropicalis*) and to food allergens (egg, peanut and cow’s milk) was carried out at the 18- and 36-month visits. All of the allergens for skin prick testing were obtained from Greer Laboratories (Lenoir, NC, USA), except for *B. tropicalis*, which was obtained from our in-house laboratory. SPTs were was taken to be positive if the wheal was at least 3 mm, and a child was considered as SPT-positive if any one or more of the individual tests was positive with a positive reaction to the positive control (histamine) and a negative reaction to the negative control (saline).

Subjects were shown pictures of eczema. Physician-diagnosed atopic eczema was based on a positive answer to the written question: “Has your child ever been diagnosed with eczema?”. “Wheezing” was based on a positive answer to the written question “Has your child ever wheezed?”, while “rhinitis” was based on a positive response to the question “Has your child ever had sneezing, running nose, blocked or congested nose, snoring or noisy breathing during sleep or when awake that has lasted for 2 or more weeks duration?” Study team members called the subjects who reported rhinitis to collect information on the number of episodes of rhinitis and the duration of each episode. A case prior to 18 months required a single episode that lasted for at least 4 weeks or two or more episodes each lasting at least 2 weeks. New cases of rhinitis after 18 months were defined by one or more episodes lasting at least 2 weeks.

Allergic clinical outcomes until 18 months were to the above-noted written questions in the first 18 months, combined with a positive SPT at 18 months. Allergic clinical outcomes until 36 months were defined as positive responses to the above-noted written questions in the first 36 months, combined with a positive SPT at 36 months. Children were included in the analysis if they were at risk for development of new allergic outcomes, i.e., did not have the allergic outcome before the period of weight gain analyzed. The allergic outcome was classified as absent when the answers for all visits were “no.” Family history of allergy was defined as positive if the mother, father or an older sibling ever had atopic eczema, asthma or allergic rhinitis.

Serial anthropometric measurements of weight at birth, 3 weeks, and 3, 6, 9 12 and 15 months were taken by trained research staff. Infant weight was recorded to the nearest gram using a calibrated infant scale (SECA 334 Weighing Scale, SECA Corp.). All measurements were taken in duplicate and the average used for all analyses.

Classification of breastfeeding has been previously described. [12] High breastfeeding was defined by exclusive or predominant breastfeeding for at least 4 months, with subsequent partial breastfeeding to at least 6 months, while low breastfeeding was defined as exclusive formula feeding or weaning before 3 months. Intermediate breastfeeding was defined as breastfeeding to at least 3 months but without meeting the criteria for high breastfeeding.

Ethics approval was obtained from the Domain Specific Review Board of Singapore National Healthcare Group and the Centralised Institutional Review Board of SingHealth. Informed written consent was obtained from all subjects.

### Statistical analysis

Statistical analysis was carried out using IBM SPSS version 20.0 (IBM SPSS Statistics, Armonk, NY). The weight change from the initial weight at the beginning of each period to the final weight at the end of each period was calculated in kilograms and divided into quartiles. The strength of association between quartiles of weight gain and the allergic outcomes was estimated using univariable and multivariable logistic regression (adjusting for relevant covariates). Chinese and male were used as the reference categories for ethnicity and sex, respectively.

## Results

### Description of the study cohort

**Fig. 1:**
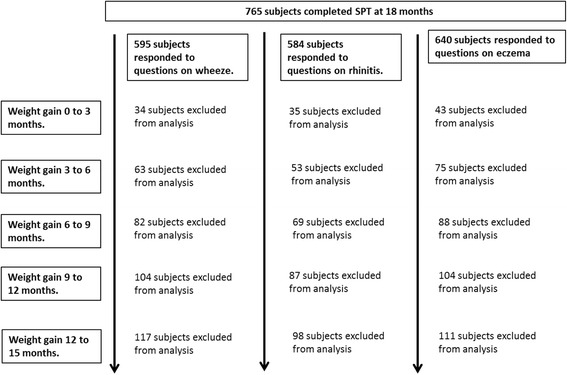
Schematic diagram of children who completed the SPT at 18 months

**Fig. 2:**
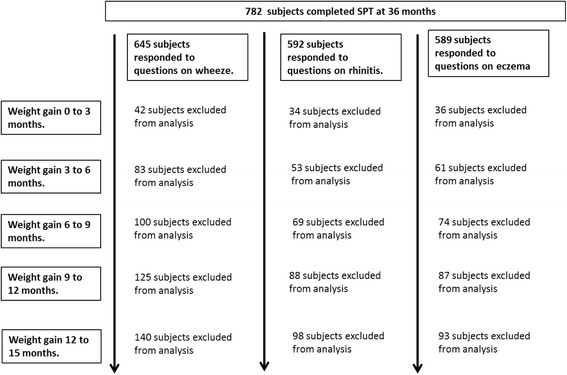
Schematic diagram of children who completed the SPT at 36 months

Of the 1247 mothers recruited into GUSTO, 1059 gave birth to full-term infants (gestational age ≥ 37 weeks) and were considered eligible for this study. The response rates to questions on allergic outcomes and SPT as well as schematic diagrams of the children included in the analysis are shown in Figs. 1 and 2. The main reason for non-completion of the questionnaires was the mothers’ not having been contactable and hence not having a home visit. Tables 1 and 2 compare the characteristics of those children with complete information and those with missing data. While the distribution of sex remains fairly similar between those with complete information and those with missing data, there are some differences in distribution of ethnicity and maternal education levels between them.

**Table 1:** Comparison of study children who completed questionnaires and SPT at 18 months vs other GUSTO children

	N(%)											
	Subjects that complete questions on rhinitis and SPT	Excluded	*P*-value	Subjects that complete questions on wheeze and SPT	Excluded	*P*-value	Subjects that complete questions on eczema and SPT	Excluded	*P*-value	Subjects that complete SPT	Excluded	*P*-value
Gender												
Male	299 (51.2)	262 (55.2)	0.2	299.(50.3)	262 (56.5)	0.047	326 (50.9)	235 (56.1)	0.1	398 (52.0)	163 (55.4)	0.3
Female	285 (48.8)	213 (44.8)		296 (49.7)	202 (43.5)		314 (49.1)	184 (43.9)		367 (48.0)	131 (44.6)	
Ethnicity												
Chinese	352 (60.3)	253 (53.3)	0.1	363 (61.0)	242 (52.2)	<0.01	387 (60.5)	218 (52.0)	<0.01	438 (57.3)	167 (56.8)	0.8
Malay	138 (23.6)	127 (26.7)		147 (24.7)	118 (25.4)		156 (24.4)	109 (26.0)		194 (25.4)	71 (24.1)	
Indian	94 (16.1)	95 (20.0)		85 (14.3)	108 (22.4)		97 (15.2)	92 (22.0)		133 (17.4)	56 (19.0)	
Maternal Education ≥12 years	371 (64.0)	267 (57.3)	0.03	375 (63.5)	263 (57.8)	0.1	401 (63.1)	237 (57.7)	0.1	464 (61.3)	174 (60.2)	0.8
Maternal Education <12 years	209 (36.0)	199 (42.7)		216 (36.5)	192 (42.2)		234 (36.9)	174 (42.3)		293 (38.7)	115 (39.8)

**Table 2:** Comparison of study children who completed questionnaires and SPT at 36 months vs other GUSTO children

	N(%)											
	Subjects that complete questions on rhinitis and SPT	Excluded	*P*-value	Subjects that complete questions on wheeze and SPT	Excluded	*P*-value	Subjects that complete questions on eczema and SPT	Excluded	*P*-value	Subjects that complete SPT	Excluded	*P*-value
Sex												
Male	303 (51.2)	258 (55.2)	0.2	342 (53.0)	219 (52.9)	1.00	304 (51.6)	257 (54.7)	0.3	414 (52.9)	147 (53.1)	1.00
Female	289 (48.8)	209 (44.8)		303 (47.0)	195 (47.1)		285 (48.4)	213 (45.3)		368 (47.1)	130 (46.9)	
Ethnicity												
Chinese	360 (60.8)	245 (52.5)	0.02	377 (58.4)	228 (55.1)	0.04	349 (59.3)	256 (54.5)	0.02	446 (57.0)	159 (57.4)	0.60
Malay	139 (23.5)	126 (27.0)		168 (26.0)	97 (23.4)		152 (25.8)	113 (24.0)		201 (25.7)	64 (23.1)	
Indian	93 (15.7)	96 (20.6)		100 (15.5)	89 (21.5)		88 (14.9)	101 (21.5)		135 (17.3)	54 (19.5)	
Maternal Education ≥12 years	379 (64.2)	259 (56.8)	0.02	395 (61.5)	243 (60.1)	0.70	365 (62.4)	273 (59.2)	0.3	474 (61.1)	164 (60.7)	0.90
Maternal Education <12 years	211 (35.8)	197 (43.2)		247 (38.5)	161 (39.9)		220 (37.6)	188 (40.8)		302 (38.9)	106 (39.3)	

A total of 103 (13.5%) subjects had a positive SPT at 18 months, of whom 82 (7.7%) had a positive SPT to inhalant allergens while 36 (3.4%) a positive SPT to food allergens and 15 (1.4%) a positive SPT to both. Twenty one (3.5%) subjects had wheeze and a positive SPT, 23 (3.9%) rhinitis and a positive SPT, and 39 (6.1%) eczema and a positive SPT.

A total of 184 children (23.5%) had a positive SPT at 36 months; 180 (17.0%) had a positive SPT to inhalant allergens, 15 (1.4%) had a positive SPT to food allergens and 11 (1.0%) a positive SPT to both. Seventy-seven(11.9%) subjects had wheeze and a positive SPT, 72 (12.2%) rhinitis and a positive SPT, and 68 (11.5%) eczema and a positive SPT.

### Associations between weight gain and allergic outcomes

As shown in Table 3, increasing weight gain quartile from 6 to 9 months, 9 to 12 months and 12 to 15 months was associated with a reduced risk of developing a positive SPT at 18 months (p-trend ≤0.03). Comparing extreme weight gain quartiles between 6 to 9 months, infants in the highest quartile had a reduced risk of a positive SPT at 18 months [adjusted odds ratio 0.3 (0.1–0.7)] compared with the lowest quartile, after adjustment for baseline weight at the beginning of the period, family history of allergy, ethnicity, sex, maternal education levels, breastfeeding, maternal height and maternal BMI. Similarly, the highest quartile of weight gain between 12 to 15 months was associated with a reduced risk of a positive SPT at 18 months vs the lowest quartile [adjusted odds ratio 0.4 (0.2–0.8)]. A similar but nonsignificant association was observed for weight gain from 9 to 12 months.

**Table 3:** Associations between infant weight gain and allergic outcomes by 18 months

		Quartile 1	Quartile 2	Quartile 3	Quartile 4	Q u arti l e 1	Quartile 2	Q uartile 3	Q u arti le 4	
Period of weight gain	Allergic outcomes	N(%)	N(%)	N(%)	N(%)	Reference group	Adjusted OR (95% CI)	Adjusted OR (95% CI)	Adjusted OR (95% CI)	p-trend
0 to 3 months	Wheeze with a positive skin prick test	1 (0.6)	5 (3.0)	2 (1.3)	11 (7.6)	1.00	1.4 (0.1–17.9)	1.3 (0.1–18.6)	4.5 (0.4–53.4)	0.2
	Prolonged rhinitis with a positive skin prick test	3 (1.9)	1 (0.7)	2 (1.4)	7 (5.2)	1.00	0.2 (0.0–3.0)	0.2 (0.0–3.4)	1.7 (0.2–14.5)	0.2
	Atopic eczema with a positive skin prick test	5 (3.0)	4 (2.4)	2 (1.3)	9 (6.2)	1.00	0.4 (0.1–2.4)	0.3 (0.0–2.2)	0.8 (0.1–4.9)	0.8
	Positive skin prick test	28 (14.1)	25 (14.0)	12 (6.7)	34 (19.3)	1.00	1.1 (0.5–2.4)	0.6 (0.2–1.4)	1.7 (0.7–3.9)	0.5
3 to 6 months	Wheeze with a positive skin prick test	5 (3.2)	4 (2.5)	2 (1.3)	3 (1.9)	1.00	1.1 (0.1–8.5)	0.5 (0.0–6.5)	1.8 (0.3–12.4)	0.7
	Prolonged rhinitis with a positive skin prick test	0 (0)	4 (2.8)	4 (2.9)	4 (2.9)	1.00	#	#	#	#
	Atopic eczema with a positive skin prick test	5 (3.1)	3 (1.8)	1 (0.7)	3 (2.0)	1.00	1.6 (0.2–10.6)	#	1.0 (0.1–8.7)	0.6
	Positive skin prick test	31 (17.6)	30 (16.8)	21 (12.4)	14 (8.3)	1.00	1.4 (0.7–3.0)	0.9 (0.4–2.0)	0.6 (0.2–1.4)	0.1
6 to 9 months	Wheeze with a positive skin prick test	3 (1.9)	2 (1.2)	5 (3.1)	3 (2.0)	1.00	0.3 (0.0–3.6)	1.6 (0.3–9.7)	0.6 (0.1–7.9)	0.9
	Prolonged rhinitis with a positive skin prick test	2 (1.4)	1 (0.7)	3 (2.1)	1 (0.8)	1.00	1.0 (0.1–12.6)	0.7 (0.1–9.6)	0.7 (0.0–11.4)	0.8
	Atopic eczema with a positive skin prick test	5 (3.2)	4 (2.6)	1 (0.7)	1 (0.7)	1.00	2.4 (0.3–16.7)	0.6 (0.0–7.0)	#	0.3
	Positive skin prick test	30 (17.9)	26 (15.4)	22 (13.2)	16 (9.7)	1.00	0.5 (0.2–1.1)	0.5 (0.2–1.0)	*0.3 (0.1–0.7)*	*<0.01*
9 to 12 m o n th s	Wheeze with a positive skin prick test	3 (1.9)	1 (0.6)	3 (1.9)	0 (0)	1.00	0.7 (0.1–9.6)	1.8 (0.2–16.1)	#	0.5
	Prolonged rhinitis with a positive skin prick test	1 (0.7)	0 (0)	0 (0)	2 (1.3)	1.00	#	#	#	#
	Atopic eczema with a positive skin prick test	3 (2.0)	2 (1.3)	1 (0.7)	3 (2.0)	1.00	0.6 (0.0–7.0)	0.4 (0.0–4.9)	#	0.2
	Positive skin prick test	34 (20.0)	26 (15.8)	12 (7.3)	20 (11.3)	1.00	0.9 (0.4–1.8)	*0.4 (0.2–0.96)*	0.5 (0.2–1.2)	*0.03*
12 to 15 months	Wheeze with a positive skin prick test	0 (0)	3 (1.7)	0 (0)	1 (0.6)	1.00	#	#	#	#
	Prolonged rhinitis with a positive skin prick test	0 (0)	0 (0)	0 (0)	0 (0)	1.00	#	#	#	#
	Atopic eczema with a positive skin prick test	1 (0.6)	0 (0)	0 (0)	2 (1.2)	1.00	#	#	#	#
	Positive skin prick test	32 (17.7)	25 (14.3)	19 (11.2)	20 (11.3)	1.00	0.7 (0.3–1.4)	0.5 (0.2–1.0)	*0.4 (0.2–0.8)*	*<0.01*

Weight gain at 0–3 months adjusted for birthweight for gestational age and sex, family history of allergy, ethnicity, sex, gestational age, breastfeeding, maternal education levels, maternal height and maternal BMI Weight gain (Kg) at 3–6 months, 6–9 months, 9–12 months and 12–15 months were adjusted for the baseline weight at the beginning of the period, family history of allergy, ethnicity, sex, breastfeeding, maternal education levels, maternal height and maternal BMI

#Not estimable, owing to insufficient number of children with studied outcomes Effects of infant weight gain on allergic outcomes

Values in italics have reached statistical significance with *p*-value <0.05

Further sub-analysis of the associations between increasing weight gain and positive SPT to inhalant allergens and to food allergens showed a similar trend. Increasing weight gain from 6 to 9 months was associated with a reduced risk of developing positive SPT to inhalant allergens, in particular to *Dermatophagoides pteronyssinus*, *Dermatophagoides farinae*(p-trend <0.05 Additional file 1: Tables S1 and S2). Increasing weight gain from 9 to 12 months was associated with a reduced risk of developing positive SPT to food allergens (p-trend =0.03, Additional file 1: Table S1).

No significant associations were observed between weight gain in any period and wheeze, allergic rhinitis or atopic eczema.

As shown in Table 4, increasing weight gain quartile from 12 to 15 months was associated with a reduced risk of developing a positive SPT at age 36 months (p-trend <0.01). Comparing extreme weight gain quartiles between 12 to 15 months, infants in the highest quartile had a reduced risk of a positive SPT at 18 months [adjusted odds ratio 0.4 (0.2–0.8)] compared with the lowest quartile. Further sub-analysis of the association between increasing weight gain and positive SPT to inhalant allergens showed a similar trend. Increasing weight gain from 12 to 15 months was associated with a reduced risk of developing positive SPT to inhalant allergens, in particular to *Dermatophagoides pteronyssinus*, *Dermatophagoides farinae* (p-trend <0.05, Additional file 1: Tables S3 and S4). Increasing weight gain from 3 to 6 months and 9 to 12 months was associated with a reduced risk of developing a positive SPT to food allergens at 36 months (p-trend <0.05, Additional file 1: Table S3).

**Table 4:** Associations between infant weight gain and allergic outcomes by 36 months

		Quartile 1	Quartile 2	Quartile 3	Quartile 4	Quartile 1	Quartile 2	Quartile 3	Quartile 4	
Period of weight gain	Allergic outcomes	N (%)	N (%)	N (%)	N (%)	Reference group	Adjusted OR (95% CI)	Adjusted OR (95% Adjusted OR CI) (95% CI)	Adjusted OR (95% Adjusted	p-trend
0 to 3 months	Wheeze with a positive skin prick test	11 (6.7)	15 (9.3)	19 (11.8)	19 (13.1)	1.00	1.6 (0.6–4.5)	1.8 (0.6–5.4)	1.3 (0.4–4.0)	0.7
	Rhinitis with a positive skin prick test	10 (6.7)	9 (6.3)	16 (10.6)	24 (17.6)	1.00	0.9 (0.3–3.1)	1.3 (0.4–4.3)	1.7 (0.5–6.0)	0.3
	Atopic eczema with a positive skin prick test	5 (3.4)	10 (6.7)	9 (6.6)	10 (7.3)	1.00	1.9 (0.5–7.1)	1.4 (0.3–5.8)	1.1 (0.3–4.9)	0.9
	Positive skin prick test	34 (17.4)	36 (20.0)	45 (24.7)	59 (31.7)	1.00	1.3 (0.7–2.6)	1.6 (0.8–3.3)	1.6 (0.8–3.3)	0.2
3 to 6 months	Wheeze with a positive skin prick test	12 (7.6)	14 (8.4)	7 (4.5)	16 (11.0)	1.00	1.3 (0.4–3.9)	0.3 (0.1–1.5)	2.6 (0.9–7.3)	0.2
	Rhinitis with a positive skin prick test	9 (6.3)	22 (14.7)	10 (7.0)	13 (10.3)	1.00	6.7 (1.4–31.8)	3.2 (0.6–16.4)	4.5 (0.9–22.2)	0.3
	Atopic eczema with a positive skin prick test	7 (4.9)	6 (4.0)	2 (1.4)	7 (5.3)	1.00	0.8 (0.2–3.1)	0.2 (0.0–1.6)	1.0 (0.3–3.8)	0.8
	Positive skin prick test	43 (23.8)	41 (22.7)	46 (25.8)	35 (21.1)	1.00	1.1 (0.6–2.2)	1.1 (0.6–2.1)	0.9 (0.5–1.8)	0.8
6 to 9 months	Wheeze with a positive skin prick test	12 (7.6)	11 (6.7)	13 (8.5)	8 (5.3)	1.00	0.4 (0.1–1.4)	0.7 (0.2–2.0)	1.1 (0.3–3.5)	0.9
	Rhinitis with a positive skin prick test	12 (8.3)	13 (9.2)	12 (8.6)	9 (7.1)	1.00	0.5 (0.1–1.8)	0.6 (0.2–2.0)	0.5 (0.1–1.9)	0.3
	Atopic eczema with a positive skin prick test	6 (4.2)	7 (5.1)	4 (3.0)	2 (1.6)	1.00	1.4 (0.4–5.8)	0.8 (0.2–3.6)	0.7 (0.1–4.3)	0.6
	Positive skin prick test	40 (23.3)	45 (25.9)	37 (22.2)	34 (20.4)	1.00	0.5 (0.3–0.96)	0.5 (0.3–1.0)	0.5 (0.3–1.0)	0.06
9 to 12 m o n th s	Wheeze with a positive skin prick test	14 (8.9)	7 (4.5)	8 (4.9)	9 (5.5)	1.00	0.5 (0.1–1.6)	0.7 (0.2–2.1)	*0.2 (0.0–0.8)*	*0.04*
	Rhinitis with a positive skin prick test	11 (8.2)	8 (6.0)	9 (6.1)	17 (11.7)	1.00	0.5 (0.1–2.3)	0.7 (0.2–2.7)	1.2 (0.3–4.2)	0.7
	Atopic eczema with a positive skin prick test	4 (2.9)	1 (0.7)	6 (4.6)	4 (2.9)	1.00	#	1.3 (0.3–5.7)	0.5 (0.1–3.1)	0.9
	Positive skin prick test	43 (24.9)	39 (23.8)	37 (21.4)	39 (22.9)	1.00	0.9 (0.5–1.6)	0.8 (0.4–1.5)	0.5 (0.3–1.1)	0.1
12 to 15 months	Wheeze with a positive skin prick test	6 (3.6)	10 (6.0)	8 (4.8)	8 (4.6)	1.00	1.7 (0.5–5.5)	0.5 (0.1–2.1)	1.1 (0.3–4.0)	0.6
	Rhinitis with a positive skin prick test	8 (6.1)	15 (10.7)	11 (7.6)	9 (6.0)	1.00	1.3 (0.3–5.2)	1.3 (0.3–5.1)	0.8 (0.2–3.3)	0.7
	Atopic eczema with a positive skin prick test	4 (2.7)	5 (3.3)	2 (1.4)	3 (1.9)	1.00	1.3 (0.3–6.3)	#	0.8 (0.1–5.4)	0.3
	Positive skin prick test	49 (27.8)	40 (23.1)	37 (20.9)	37 (20.6)	1.00	0.6 (0.3–1.0)	*0.4 (0.2–0.8)*	*0.4 (0.2–0.8)*	*<0.01*

Weight gain at 0–3 months adjusted for birthweight for gestational age and sex, family history of allergy, ethnicity, gestational age, sex, breastfeeding, maternal education levels, maternal height and maternal BMI. Weight gain (Kg) at 3–6 months, 6–9 months, 9–12 months and 12–15 months were adjusted for the baseline weight at the beginning of the period, family history of allergy, ethnicity, sex, breastfeeding, maternal education levels, maternal height and maternal BMI

#Not estimable, owing to insufficient number of children with studied outcomes

Values in italics have reached statistical significance with *p*-value <0.5

No significant associations were obtained between weight gain in any period and allergic wheeze, allergic rhinitis or atopic eczema by 36 months.

## Discussion

Rapid weight gain in the first year of life among GUSTO children was associated with a reduced risk of allergen sensitization at age 18 months. Weight gain from 12 to 15 months of life reduced the risk of allergen sensitization at both 18 and 36 months. Findings from previous studies have been mixed. The PROBIT study from Belarus found no consistent associations between infant weight gain and SPT results at 6.5 years but observed an inverse association of weight gain velocity from 12 to 34 months and from 34 to 60 months. [7] In the United Kingdom, 1548 children were followed up with SPTs at 3 years; no significant associations were observed with postnatal weight gain velocity. [9] Similarly, the PIAMA birth cohort study from the Netherlands followed a subgroup (*n* = 1554) to 8 years and found no associations between BMI changes from 1 to 2 years and allergen-specific IgE at 8 years. [8] Finally, the SCAALA cohort study from Brazil reported a lower mean z-score for growth rate in the first 2 years of life among non-sensitized (SPT-negative) children aged 4–11 years. [6].

A possible reason for the inconsistent results of these studies is the earlier age at which SPTs were obtained in the GUSTO cohort: 18 and 36 months in our study vs 3–11 years in other cohorts. Allergen sensitization patterns are known to change with age. [13, 14] The association we observed between increased weight gain and reduced subsequent allergen sensitization may be a chance finding, however, and requires confirmation in other studies. While our observations are in agreement with studies reporting an increased sensitization to food allergens in underweight individuals, [15] other studies have reported increased food allergen sensitization in overweight individuals vs those of normal weight. [16, 17] If confirmed, one possible mechanism for the associations we observed is increased leptin secretion from adipose tissue, which could skew the immune response towards a T-helper type 1 (Th1) response, with subsequent production of pro-Th1 cytokines such as IFN-γ and IL-2 and suppressed production of pro-T-helper 2 (Th2) cytokines such as IL-4, [18–20] thereby reducing allergen sensitization.

We observed a nonsignificant positive association between weight gain from 0 to 3 months and allergic wheeze (i.e., wheeze with a positive SPT) by 18 months, which is limited by the small number of subjects with allergic wheeze. An association between increasing weight gain in the first 3 months and risk of wheeze has been reported in several previous studies. [7, 21] The PROBIT study from Belarus reported that weight gain velocity between 0 to 3 months was positively associated with ever having wheezed by 6.5 years. [7] Similarly, a study from the United Kingdom found a 1-SD increase in weight gain from birth to 6 months to be associated with a statistically significant 22% increase in risk of atopic wheeze (defined as ever having wheezed by 3 years and a positive SPT at 3 years). [9].

An important strength of our study is its prospective collection of outcome data at multiple time points. A limitation, however, is that allergic symptoms were all reported by the parent (usually the mother). We therefore used the SPT as an objective assessment of allergic sensitization, both alone and in combination with common symptoms and diagnoses that may have an allergic etiology. Another limitation is low statistical power, owing to missing data from non-completion of questionnaires. It will be important to track the future development of allergic diseases and immune phenotypes in our cohort to assess whether the associations we observed persist, and whether new ones emerge at later ages.

## Conclusion

Higher weight gain in the first 15 months of life was associated with a reduced risk of allergen sensitization, but not with combinations of allergic symptoms. The dissociation between SPT and clinical symptoms could be due to the less specific nature of clinical symptoms of rash, rhinitis and wheezing which could be of non-atopic origins such as viral induced.

## Additional file

Additional file 1: Table S1. Associations between infant weight gain and positive skin prick test to inhalant and food allergens by 18 months. **Table S2.** Associations between infant weight gain and positive skin prick test to individual allergens by 18 months. **Table S3.** Associations between infant weight gain and positive skin prick test to inhalant and food allergens by 36 months. **Table S4.** Associations between infant weight gain and positive skin prick test to individual allergens by 36 months. (DOCX 63 kb)

## Abbreviations

CI: Confidence interval
OR: Odds ratio
SPT: Skin prick test
