# Supplementary material for: Effects of infant weight gain on subsequent allergic outcomes in the first 3 years of life
Source: BMC Pediatr. 2017 Jun 2;17:134. doi: 10.1186/s12887-017-0890-0 (PMC5457600; doi:10.1186/s12887-017-0890-0)
Supplement: Additional file 1: Table S1. — Associations between infant weight gain and positive skin prick test to inhalant and food allergens by 18 months. Table S2. Associations between infant weight gain and positive skin prick test to individual allergens by 18 months. Table S3. Associations between infant weight gain and positive skin prick test to inhalant and food allergens by 36 months. Table S4. Associations between infant weight gain and positive skin prick test to individual allergens by 36 months. (DOCX 63 kb) [file 12887_2017_890_MOESM1_ESM.docx]

**Additional file 1: Table S1 Associations between infant weight gain and positive skin prick test to inhalant and food allergens by 18 months**

* Weight gain at 0-3 months adjusted for birthweight for gestational age and sex, family history of allergy, ethnicity, sex, gestational age, maternal education levels, breastfeeding, maternal height and maternal BMI. Weight gain (Kg) at 3-6 months, 6-9 months, 9-12 months and 12-15 months were adjusted for the baseline weight at the beginning of the period, family history of allergy, ethnicity, sex, maternal education levels, breastfeeding, maternal height and maternal BMI.

**Additional file 1: Table S2 Associations between infant weight gain and positive skin prick test to individual allergens by 18 months**

* Weight gain at 0-3 months adjusted for birthweight for gestational age and sex, family history of allergy, ethnicity, sex, gestational age, maternal education levels, breastfeeding, maternal height and maternal BMI. Weight gain (Kg) at 3-6 months, 6-9 months, 9-12 months and 12-15 months were adjusted for the baseline weight at the beginning of the period, family history of allergy, ethnicity, sex, maternal education levels, breastfeeding, maternal height and maternal BMI.

**Additional file 1: Table S3 Associations between infant weight gain and positive skin prick test to inhalant and food allergens by 36 months**

* Weight gain at 0-3 months adjusted for birthweight for gestational age and sex, family history of allergy, ethnicity, gestational age, sex, maternal education levels, breastfeeding, maternal height and maternal BMI. Weight gain (Kg) at 3-6 months, 6-9 months, 9-12 months and 12-15 months were adjusted for the baseline weight at the beginning of the period, family history of allergy, ethnicity, sex, maternal education levels, breastfeeding, maternal height and maternal BMI.

# Not estimable, owing to insufficient number of children with studied outcomes

**Additional file 1: Table S4 Associations between infant weight gain and positive skin prick test to individual allergens by 36 months**

* Weight gain at 0-3 months adjusted for birthweight for gestational age and sex, family history of allergy, ethnicity, gestational age, sex, maternal education levels, breastfeeding, maternal height and maternal BMI. Weight gain (Kg) at 3-6 months, 6-9 months, 9-12 months and 12-15 months were adjusted for the baseline weight at the beginning of the period, family history of allergy, ethnicity, sex, maternal education levels, breastfeeding, maternal height and maternal BMI.

# Not estimable, owing to insufficient number of children with studied outcomes
